# A Cross-Sectional Study of Hyponatremia Associated with Acute Central Nervous System Infections

**DOI:** 10.3390/jcm8111801

**Published:** 2019-10-27

**Authors:** Andy K.H. Lim, Sahira Paramaswaran, Lucy J. Jellie, Ralph K. Junckerstorff

**Affiliations:** 1 General Medicine, Monash Health, Clayton, VIC 3168, Australia; sahirapw@gmail.com (S.P.); Lucy.jellie@monashhealth.org (L.J.J.); Ralph.junckerstorff@monashhealth.org (R.K.J.); 2 Infectious Diseases, Monash Health, Clayton, VIC 3168, Australia; 3 Monash University Department of Medicine, Clayton, VIC 3168, Australia

**Keywords:** sodium, hyponatremia, water-electrolyte imbalance, central nervous system infection, encephalitis, meningitis, meningoencephalitis, microbiology, herpes simplex

## Abstract

Hyponatremia can occur with central nervous system (CNS) infections, but the frequency and severity may depend on the organism and nature of CNS involvement. In this cross-sectional study at a large Australian hospital network from 2015 to 2018, we aimed to determine the prevalence and severity of hyponatremia associated with CNS infection clinical syndromes, and the association with specific organisms. We examined the results of cerebrospinal fluid analysis from lumbar punctures performed in 184 adult patients with a serum sodium below 135 mmol/L who had abnormal cerebrospinal fluid analysis and a clinical syndrome consistent with an acute CNS infection (meningitis or encephalitis). Hyponatremia affected 39% of patients and was more severe and frequent in patients with encephalitis compared to meningitis (odds ratio = 3.03, 95% CI: 1.43–6.39, after adjusting for age). Hyponatremia was present on admission in 85% of cases. Herpes simplex virus infection was associated with the highest odds of hyponatremia (odds ratio = 3.25, 95% CI: 1.13–7.87) while enterovirus infection was associated with the lowest (odds ratio = 0.36, 95% CI: 0.14–0.92), compared to cases without an isolated organism. We concluded that the risk of hyponatremia may vary by the organism isolated but the clinical syndrome was a useful surrogate for predicting the probability of developing hyponatremia.

## 1. Introduction

Acute central nervous system (CNS) infections are neurological emergencies which require prompt and effective treatment to avoid morbidity and mortality. Clinically, CNS infections may take the form of meningitis, encephalitis or brain abscesses. The cases with overlapping clinical features of meningeal irritation and brain parenchymal involvement are sometimes referred to as meningoencephalitis. The majority of CNS infections are community-acquired but some patients may develop healthcare-associated meningitis in the setting of trauma or intracranial cerebrospinal fluid shunts and external drains.

The organisms commonly isolated in adult cases of community-acquired bacterial meningitis include *Neisseria meningitidis*, *Streptococcus pneumoniae*, *Haemophilus influenzae* and in older adults, *Listeria monocytogenes*. Viral meningitis is more common than bacterial meningitis and are typically due to enteroviruses [1]. Although many organisms, including bacterial, viral, fungal and protozoans, are capable of infecting brain tissue leading to encephalitis, the most common are viral, in particular, herpes simplex virus (HSV). However, there is geographic variation in the epidemiology of causative agents. For example, Japanese encephalitis virus is prominent in Thailand [2] and CNS infection with *Mycobacterium tuberculosis* is a consideration in endemic parts of the world such as India [3]. *Cryptococcus* species are the most common fungal organism implicated in CNS infections, particular in immunocompromised patients [4]. Within the last decade, it has been recognized that encephalitis occurs just as frequently as purulent meningitis [5].

One of the causes of morbidity and mortality associated with CNS infection is hyponatremia, defined as a serum sodium below 135 mmol/L. There has been a theoretical and observed risk of hyponatremia in acute CNS infection. Originally described in the 1950s and 1960s, hyponatremia in the setting of CNS injury was attributed to either a syndrome of inappropriate antidiuretic hormone (SIADH) or cerebral salt wasting [6,7,8]. Less commonly, diabetes insipidus has been implicated [9], and a role for natriuretic peptides in promoting salt loss has also been suggested [10]. Acute severe hyponatremia is associated with neurological dysfunction due to cerebral swelling [11]. There are two potential consequences of hyponatremia in patients with CNS infection. Firstly, it may lead to a wrong or delayed diagnosis of CNS infection if neurological symptoms are attributed to the electrolyte disorder. Secondly, hyponatremia can exacerbate the neurological manifestations of the CNS infection. In any case, hyponatremia portends a higher mortality for hospitalized patients in most epidemiological studies [12,13,14,15].

The overall incidence of hyponatremia associated with CNS infection has been reported as 30%–66% [16,17,18,19]. The variation in estimates may be partly due to the clinical syndrome involved (meningitis vs. encephalitis). For example, patients with overt neurological involvement in CNS infections have a higher incidence of hyponatremia than those with systemic symptoms but no alterations in Glasgow Coma Scale (GCS), focal weakness, movement disorder or changes in reflexes [17]. The propensity for specific organisms for causing hyponatremia is another possibility for the observed variability in incidence. For example, tick-borne CNS infections were associated with a higher incidence of hyponatremia than non-tick-borne infections [20]. Therefore, it is relevant to examine whether the clinical presentation or the organism involved can be correlated to the risk of hyponatremia so that we may stratify patients at high risk for intensive biochemistry monitoring. This may also influence the nature and quantity of parenteral fluids administered. On the other hand, the finding of a low prevalence may provide justification to minimize biochemistry tests.

This study aimed to determine the prevalence and severity of hyponatremia in patients with acute CNS infection, and the association of hyponatremia with the clinical syndrome (meningitis or encephalitis) and specific infecting organism. 

## 2. Methods

### 2.1. Study Design, Setting and Patient Selection

This was a cross-sectional study conducted at the Monash Health hospital network in the state of Victoria, Australia. Monash Health is a tertiary referral center located in the south-east region of Melbourne. It is the largest health service in Victoria, and services approximately one quarter of the population of Melbourne. Eligible patients were identified from a centralized laboratory database of cerebrospinal fluid samples processed between 1 July 2015 and 30 June 2018. Only adult patients (18 years and above) were included. 

The electronic medical and laboratory records for all consecutive patients were examined to determine the reason for cerebrospinal fluid testing, to classify the infection by clinical syndrome, determine the organism responsible, and to profile the serum sodium levels. The following were exclusion criteria: lumbar puncture performed to investigate non-infectious pathology such as neurological disease (e.g., multiple sclerosis), malignancy, subarachnoid hemorrhage or perioperative sampling of intracranial shunts and drains; normocellular cerebrospinal fluid where infection has been excluded; repeated cerebrospinal fluid samples for the same patient (only the index admission results used); and patients with existing chronic hyponatremia.

### 2.2. Variables and Definitions

#### 2.2.1. Hyponatremia

Hyponatremia was the main outcome variable of interest. It was defined as a serum sodium of less than 135 mmol/L on at least 2 samples taken greater than 12 h apart. For the classification of severity, we used the nadir serum sodium. Mild, moderate and severe hyponatremia were defined as serum sodium 130 to 134 mmol/L, 120 to129 mmol/ L, and less than 120 mmol/L respectively. In the setting of hyperglycemia in diabetic patients, a correction factor for serum sodium of +1.6 mmol/L was applied for every 5.6 mmol/L increase in blood glucose above the upper limit of the reference range.

#### 2.2.2. CNS Infection

Acute CNS infection was dichotomized into two syndromes for analysis: (1) meningitis syndrome (headache, photophobia and neck stiffness), and (2) encephalitis syndrome (confusion, altered sensorium, focal neurological deficits or seizures). The criteria for defining CNS infection and encephalitis in this study was adapted from the recommendations of the International Encephalitis Consortium [21]. Probable CNS infection was defined as the presence of a clinical syndrome (symptoms and clinical signs) compatible with CNS infection, fever, elevated cerebrospinal fluid leucocyte count above 5 × 10^6^/L; and if available, supported by neuroimaging evidence of CNS infection or electroencephalogram abnormalities consistent with encephalitis. A cerebrospinal fluid leucocyte count greater than 5 × 10^6^/L was our microbiology laboratory definition of pleocytosis. A prospective diagnostic accuracy study has shown that cerebrospinal fluid leucocytosis was the best single marker which differentiated CNS infection from other diagnosis, with an area under receiver operating curve of 0.94. Using a cut-off of 5 leucocytes/µL in the cerebrospinal fluid, the reported sensitivity was 94% and specificity was 68% [22]. Confirmed CNS infection required the same criteria as *probable* infection, in addition to finding pathologic, microbiologic, or serologic evidence of acute infection with an organism associated with CNS infection. For this study, we accepted cases of probable and confirmed CNS infection. In cases where meningoencephalitis was suspected, the opinion of the infectious diseases team was considered but most were classified as encephalitis syndrome.

#### 2.2.3. Other Variables

Potential confounders of the association between hyponatremia and the infecting organism or clinical syndrome were identified based on literature review. The comorbidities of interest were diabetes, congestive heart failure, cirrhosis and chronic kidney disease. The use of commonly prescribed medications which could potentially contribute to hyponatremia were categorized into antiepileptics (e.g., valproate, carbamazepine, levetiracetam), antidepressants such as the selective serotonin reuptake or serotonin-noradrenaline reuptake inhibitors (e.g., fluoxetine, sertraline, desvenlafaxine, duloxetine) and tricyclic antidepressants (e.g., amitriptyline), antipsychotics (e.g., olanzapine, risperidone, quetiapine), diuretics (e.g., thiazides), and angiotensin system inhibitors (e.g., ramipril, candesartan). We specifically collected data on acyclovir treatment, given its potential association with hyponatremia [23]. 

To examine the contribution of intravenous fluid therapy to hyponatremia, we considered the recommendations of the National Institute for Health and Care Excellence (NICE). The NICE guidelines recommend a daily volume of 25–30 mL/kg for maintenance fluids (i.e., approximately 2 liters daily for a 70 kg person) and also suggest that prescribing more than 2.5 liters of hypotonic fluid (i.e., sodium chloride 0.18% in 4% dextrose) increases the risk of hyponatremia [24]. As a conservative approach, we considered the use of more than two liters of hypotonic fluids (e.g., 5% dextrose, sodium chloride 0.18% in 4% dextrose) per day within 48 h of developing hyponatremia as a potential confounder. Acute kidney injury was defined as a binary variable using the creatinine criteria from the Kidney Disease Improving Global Outcomes recommendations [25]. Patients with an increase in serum creatinine of 26 µmol/L (0.3 mg/dL) within 48 h, or at least 50% from baseline within seven days were considered as having acute kidney injury. The urine criteria to define acute kidney injury could not be reliably applied in this retrospective study.

### 2.3. Statistical Analysis 

We used a chi-squared analysis for comparing proportions between categorical variables, or a two-tailed Fisher’s exact test if cell counts were five or less. To compare the age distribution between the infection syndrome groups, we used the non-parametric Mann–Whitney U test due to the significant positive skew. Logistic regression was used to test the association between predictor variables and hyponatremia as a binary outcome. From the univariable analysis, we initially included all variables with a *p* < 0.20 into a base multivariable model and used a backwards elimination approach for selecting variables. Nested models were compared with likelihood ratio tests and non-nested models with Information Criteria (Akaike’s and Bayesian). The model residuals were used to examine for outliers and influential observations were identified by the delta-beta method (Pregibbon’s). All analyses were performed with STATA 15.1 (StataCorp, TX, USA). A *p* value < 0.05 was considered statistically significant. 

### 2.4. Ethics Approval and Consent

This study was approved by the Monash Health Human Research Ethics committee (RES-18-0000-501Q), an institutional review board. Patient consent was waived as only de-identified data was used for the analysis and publication.

## 3. Results

### 3.1. Patient Characteristics

**Figure 1:**
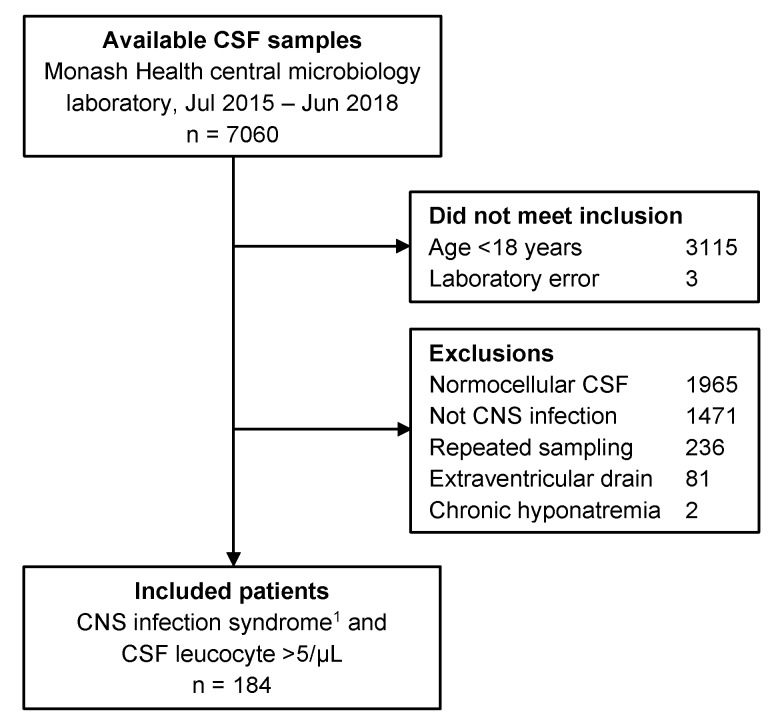
Study flow diagram detailing reasons for exclusion of cerebrospinal fluid (CSF) samples. The majority of exclusions were due to normal CSF leucocyte counts, followed by CSF testing for reasons other than central nervous system (CNS) infection (e.g., neurological disease). ^1^ meningitis or encephalitis.

The flowchart in Figure 1 outlines the outcomes of the search strategy, exclusions and the final numbers of patients included in the analysis. Table 1 summarizes the characteristics of the included patients by hyponatremia status. The patients were mostly young with few comorbidities, with only 16% (29/184) over 65 years of age. Appendix A summarizes the characteristics of the patients by infection syndrome. An encephalitis syndrome was present in 25% (46/184) of patients, while the others manifested a meningitis syndrome. Patients with encephalitis syndrome were older than patients with meningitis syndrome (*p* < 0.001), and had a higher proportion of diabetics (χ^2^ = 6.7, df = 1, *p* = 0.01), greater use of diuretics (χ^2^ = 7.8, df = 1, *p* = 0.005), antidepressants (χ^2^ = 5.6, df = 1, *p* = 0.018) and antiepileptic drugs (χ^2^ = 6.9, df = 1, *p* = 0.009). Overall, there were only few patients with congestive heart failure, significant chronic kidney disease or acute kidney injury. There were no patients with known cirrhosis. 

### 3.2. Microbiology

A specific infectious organism was isolated from 54% (99/184) of patients with clinical evidence of a CNS infection and abnormal cerebrospinal fluid composition (Table 2). Two thirds of these were due to viruses, with enteroviruses being the most frequently implicated organism. The majority of the infectious organisms were detected by a polymerase chain reaction or culture of the cerebrospinal fluid (91/99). The remainder were diagnosed by blood culture, serology and polymerase chain reaction of non-cerebrospinal fluid samples. 

### 3.3. Cerebrospinal Fluid Composition

The cerebrospinal fluid analysis is summarised in Appendix B. Bacterial infections were associated with a significantly higher cerebrospinal fluid total leukocyte count (predominantly polymorphs), lactate and protein content. On average, cerebrospinal fluid glucose levels were low in bacterial infections but relatively normal with other infections. In patients where no organism has been isolated, the pattern of cerebrospinal fluid abnormalities closely resembles that of a viral infection biochemically but with a lower cell count. It is likely that these represent milder cases of a viral CNS infection. 

### 3.4. Hyponatremia

Hyponatremia was common, affecting 39.1% (72/184) of patients in this study. Hyponatremia was mild in 73.6% (53/72), moderate in 23.6% (17/72) and severe in 2.8% (2/72) of patients. In patients with hyponatremia, this was present on admission in 84.7% (61/72) of cases. Without taking into account specific organisms, there were only small differences in the severity of hyponatremia between the organism categories of CNS infection (Table 3). It is also evident that hyponatremia was more frequent and severe in patients with encephalitis syndrome compared to meningitis syndrome (Table 4).

### 3.5. Logistic Regression

Table 5 summarises the univariable logistic regression analysis. There were too few patients with heart failure or cirrhosis for reliable estimates. Similarly, advanced chronic kidney disease was uncommon. Only 6.5% of patients had an eGFR less than 60 mL/min/1.73 m^2^. Therefore, we could not analyse risk for those with advanced chronic kidney disease (eGFR below 30 mL/min/1.73 m^2^). Cerebrospinal fluid content was not associated with hyponatremia but the opening pressure was inconsistently recorded and was not analysed. HSV infection was associated with the highest odds and enterovirus infection was associated with the lowest odds of hyponatremia. There was strong evidence that an encephalitis syndrome was associated with higher odds of hyponatremia than meningitis syndrome. 

In the univariable logistic regression, patients who used one or more medications reported to cause hyponatremia had higher odds of hyponatremia compared to patients who did not use any of these medications. However, due to the low numbers in each medicine category and the wide confidence intervals, we could not demonstrate an association between the specific categories of medications and hyponatremia. There were 79 patients who received three or more doses of acyclovir, while two patients received valganciclovir and another received valacyclovir. The majority of patients (85%, 61/72) experienced hyponatremia prior to the commencement of acyclovir; therefore, hyponatremia cannot be attributed to acyclovir in these cases. Of the patients who developed hyponatremia after admission, 56% (6/11) had received acyclovir. When the patients with admission hyponatremia were excluded, there was no evidence that acyclovir treatment was associated with hyponatremia.

As noted, hyponatremia was community-acquired in 85% of cases. Thus, we found that the use of hypotonic or saline-free fluids was fairly limited, in terms of frequency and volume. Only one patient received significant amounts of hypotonic fluids and he did not develop hyponatremia. Thus, excessive hypotonic fluid administration was not shown to be a risk factor for hyponatremia in our study. The prevalence of acute kidney injury was low at 6.5% but there was weak evidence that AKI was associated with hyponatremia (*p* = 0.054).

#### 3.5.1. Multivariable Logistic Regression Model

Two possible approaches for modelling were possible, one using the clinical syndrome as the main explanatory factor, and the other using the organism. If both variables were included in a multivariable model, only clinical syndrome was statistically significant. This is likely because the clinical syndrome is partly determined by the organism. In the multivariable models, age was not independently associated with hyponatremia but it was a confounder of the effect of infection syndrome or organism on hyponatremia (−20% in beta-coefficient). The occurrence of acute kidney injury (−2% in beta-coefficient) and the use of medications associated with hyponatremia (−3% in beta-coefficient) did not confound the regression estimates for the main explanatory variable and were also not statistically significant in the multivariable models and were not included in the final model. The likelihood ratio tests also confirmed that neither acute kidney injury (LRχ^2^ = 1.92, *p* = 0.17) nor medications (LRχ^2^ = 0.79, *p* = 0.37) contributed additional information to the models. 

A parsimonious clinical model included age and CNS infection syndrome (Table 6). On average, the encephalitis syndrome was associated with three-fold higher odds of hyponatremia compared to a meningitis syndrome, after adjusting for patient age. A comparison of the two models with information criteria showed that the model using CNS infection syndrome was a better fit for the data than a model using organism information (Akaike’s 235.4 vs. 239.4; Bayesian 245.0 vs. 274.4).

#### 3.5.2. Sensitivity Analysis

Our definition of cerebrospinal fluid pleocytosis was conservative and optimised for high sensitivity. We considered the potential impact of a higher leucocyte cut-off of 10 cells/µL on our results. However, only seven patients (3.8%) included in our study had cerebrospinal fluid leucocytes less than 10 cells/µL. Of these seven, two patients had organisms isolated (one HSV-1 and one enterovirus). We performed a sensitivity analysis by excluding the five patients with cerebrospinal fluid leucocyte counts between 5 and 10 cells/µL which were isolate-negative. There was only a small change in our logistic regression model estimates reported in Table 6. The odds ratio for encephalitis syndrome increased to 3.37 (95% CI: 1.56–7.29, *p* = 0.002), which is an 11% increase. Thus, the estimated effect was larger rather than diminished.

## 4. Discussion

Hyponatremia is the most common electrolyte abnormality in hospitalized patients, especially those with critical neurological injuries (affecting up to 60%) and is associated with increased mortality and hospital length of stay [26,27,28]. In this cross-sectional study at a large hospital network, we examined cerebrospinal fluid results in patients with CNS infection and confirmed that hyponatremia is a common manifestation of CNS infection, affecting 39% of cases. Thus, hyponatremia is not limited to patients with brain malignancy, neurotrauma, or neurosurgery. We also demonstrated an association between hyponatremia and CNS infection with HSV and enteroviruses, and an association between hyponatremia and the clinical syndrome of meningitis or encephalitis at presentation.

Historically, most studies have focused on assessing the incidence and impact of hyponatremia in neurosurgical cohorts [26,27,28,29]. Studies of hyponatremia and CNS infection have mainly been concerned about HSV infection [30] or examined encephalitis [17] and meningitis [16,31] separately. Not many studies have compared meningitis and encephalitis in the same study. Our 39% prevalence of hyponatremia provided an overall estimate, which varied depending on the organism or clinical syndrome. In this respect, HSV infection was associated with the highest risk for hyponatremia with a prevalence of 68%, and enteroviruses with the lowest (19%) in our study. This finding is consistent with previous studies. In a study of viral encephalitis, patients infected by HSV-1 had an almost three-times higher percentage of hyponatremia compared to non-HSV-1 viral infections (56.3% vs. 19.6%). The authors suggested that hyponatremia may be a useful marker for HSV-1 CNS infection [19]. Other studies have shown rates of hyponatremia of 43%–55% in patients with HSV-1 encephalitis [30,32]. Thus, while viral CNS infections showed an overall lower prevalence of hyponatremia than bacterial infections in our study, this was due the fact that enteroviruses were the most common viral infections and have the lowest prevalence of hyponatremia. 

Other than HSV studies, there are reports indicating that the organism responsible for the CNS infection matters with regard to hyponatremia. Czupryna et al. reported a higher frequency of hyponatremia in tick-borne encephalitis compared to non-tick-borne viral meningitis [20]. We noted a signal for a higher odds of hyponatremia with *Streptococcus pneumoniae* CNS infection, but the confidence interval was wide due to the small numbers. In a study of community-acquired meningitis, Brouwer et al. showed that hyponatremia varied with the infecting organism. The authors found that *S. pneumoniae* meningitis had the higher prevalence of hyponatremia (33%) compared to *Neisseria meningitidis* (21%), a finding which was hinted at in our data. Others have found a prevalence of hyponatremia as high as 73% with tuberculous meningitis [31]. We could not convincingly demonstrate these additional associations, but this may be due to a type 2 error.

There was a tendency for patients with HSV encephalitis to be older, as seen in previous studies [19]. In our regression model, we adjusted for age as we detected evidence of confounding (age was associated with both encephalitis and hyponatremia). Furthermore, all the enterovirus cases presented with a meningitis syndrome while all HSV cases had an encephalitis syndrome. Therefore, it made sense not to include both organism and clinical syndrome in the same model because of this correlation. Instead, we showed that the clinical syndrome itself is just as predictive, and patients with encephalitis syndrome had twice the prevalence of hyponatremia than patients with meningitis syndrome. This may be more useful clinically given that the specific organism could not be isolated from the cerebrospinal fluid or other microbiological means in 37%–50% of cases of CNS infection [1,33,34]. In our study, an organism could not be isolated in 46% of cases. Hence, a typical encephalitis syndrome was associated with three-fold higher odds of hyponatremia compared to meningitis syndrome.

This study is not about causality. Hyponatremia and the CNS infecting organism could be associated in a bidirectional manner. The relevance of detecting hyponatremia may include: (1) raising the index of suspicion for HSV encephalitis and prompting a lumbar puncture, which may not have been considered otherwise, (2) influencing or justifying the empirical treatment with acyclovir in patients with delayed sampling of the cerebrospinal fluid, and (3) incorporation into prediction rules for estimating the probability of HSV involvement, as performed by Gennai et al. [35]. On the other hand, the presenting clinical syndrome of encephalitis could suggest a higher risk of concurrent hyponatremia. Hyponatremia portends a worse prognosis both for morbidity and mortality in multiple studies of diverse populations and conditions. Thus, this clinical information may be useful in determining the initial choice of intravenous fluids prior to availability of the serum sodium results or the intensity of biochemistry monitoring in the smaller subgroup of patients with onset of hyponatremia after admission.

It is worth discussing the negative findings from this study. Firstly, we found no evidence that medications contributed to hyponatremia despite case reports implicating antidepressants, antipsychotics, antiepileptics, diuretics and angiotensin system inhibitors [36]. Much like comorbidities, the frequency of medication use was low. In particular, only eight patients were on thiazide diuretics or spironolactone. Fourteen patients were on furosemide, which is rarely a cause of hyponatremia given its action as a loop diuretic. We specifically examined the use of acyclovir, given case reports that it may contribute to hyponatremia [23,37]. The majority of patients with hyponatremia were diagnosed on presentation, prior to acyclovir treatment. When these patients were excluded, we found no association between acyclovir and hyponatremia. However, we cannot prove that hyponatremia was exacerbated by acyclovir. Secondly, we found no association between cerebrospinal fluid cell count or biochemistry and hyponatremia. Theoretically, the cerebrospinal fluid abnormalities would correlate with the severity of infection and hence the probability of complications. In the community-acquired meningitis study, the cerebrospinal fluid leucocyte count and protein level was higher in those with hyponatremia than those without [16]. However, this was a much-restricted study population compared to ours. In encephalitis patients, HSV-1 viral load in the cerebrospinal fluid did not predict outcomes, although red cell count was a possible poor prognostic indicator [38]. This may be more relevant to meningitis syndrome than encephalitis syndrome.

This study suggests an appropriate use of intravenous fluids overall, without evidence for excessive hypotonic fluids. This may be due to the fact that most cases of hyponatremia were obvious at presentation or that appropriate guidelines were followed [24]. The patients were also relatively younger than most general medical hospital patients, with few comorbidities predisposing to hyponatremia. Thus, we believe that hyponatremia is unlikely to be iatrogenic in our study. However, our definition of “excessive” was arbitrary although based on general recommendations for maintenance of intravenous fluids per NICE guidelines. 

The generalizability of the study results depends on the local epidemiology. It is pertinent in areas where HSV encephalitis is the predominant organism responsible for encephalitis such as Australia and most of the United Kingdom. Knowledge of the local epidemiology would be needed before generalizing these results. With regards to the implications for practice, we recommend a prompt assessment of serum sodium in patients presenting with encephalitis syndrome, and 0.9% sodium chloride should be considered as the initial choice of intravenous fluid. Detection of hyponatremia in a patient with suspected CNS infection should prompt consideration of HSV infection, with immediate action to confirm the diagnosis or initiate acyclovir therapy if delays are anticipated.

### 4.1. Study Limitations

Given the cross-sectional design of the study, we could not infer a direct causation of hyponatremia by the CNS infection. As discussed, there may be multiple mechanisms and contributory factors, some of which may remain unaccounted for. Thus, residual confounding may remain in retrospective studies such as ours. However, cohort studies would be near impossible to conduct given that acute CNS infections occur unpredictably and progress quickly, such that hyponatremia is often present on admission. Cross-sectional studies may be the only feasible option. The frequency of infection by certain organisms was too low for reliable estimates in our logistic regression models. The patients in this study were relatively young and had little comorbidities compared to the general hospital population of acutely ill adults. Thus, we were unable to assess the full spectrum of comorbidities associated with the risk of hyponatremia, such as cirrhosis and heart failure. This study did not look at clinical outcomes, such as mortality, given the low inpatient mortality rate of 1.1% for the study period. We suspect that hyponatremia was also associated with an increased hospital length of stay, as previously shown, but we did not specifically address this in our study [27]. 

Using a cerebrospinal fluid leucocyte count above 5 cells/µL to define pleocytosis could have biased our estimates if the specificity for CNS infection was low at this threshold. One prospective study of cerebrospinal fluid analysis from undifferentiated patients showed that the sensitivity and specificity for CNS infection at this cut-off was 94% and 68%, respectively. Using a threshold of 1000 leucocytes/µL, the sensitivity was 13% and the specificity increased to 100% [22]. Another study indicated that the number of differential diagnosis was larger with leucocyte counts below 50 cells/µL [39]. We feel justified using a threshold of 5 leucocytes/µL as our patients were not undifferentiated. Given the retrospective analysis, our patients underwent diagnostic workup and patients with a diagnosis other than CNS infection were excluded (see Figure 1). Our sensitivity analysis suggests that using a threshold of 10 leucocytes/µL would not have altered our conclusion and we would have missed two cases of confirmed CNS infection. In another study, 10% of patients with CNS infection had cerebrospinal fluid leucocytes counts of 6–10 cells/µL [39].

### 4.2. Research Recommendations

We suggest that future studies of CNS infections consider assessing for hyponatremia, in terms of etiology and clinical outcomes. Studies designed to evaluate prediction models for HSV CNS infection should include the serum sodium as a potential independent variable. Studies of the less common organisms and their association with hyponatremia are needed. Given their infrequent nature, collaborations between health services are needed. Pediatric patients have a different susceptibility and risk for dysnatremia, which warrants further investigation. As technology and microbiological science improve, many of the currently isolate negative cerebrospinal fluid samples may turn out to be due to previously unrecognized infectious agents, and these associations should be periodically reviewed in the future.

Although there are many potential causes of hyponatremia, two specific conditions need consideration in the setting of CNS infection. These are SIADH and cerebral salt wasting. Our study was not set up to determine the mechanism of hyponatremia. This would require all patients to have sodium and osmolality profiles determined in the blood and urine and a thorough clinical assessment of volume status. Previous studies have reported that either SIADH or cerebral salt wasting was possible in a CNS infection [6,7,8,10]. However, there is currently no reliable data to indicate which mechanism dominates and whether it depends on the clinical syndrome (encephalitis or meningitis) or the specific organism. With further data in this area, clinicians may be able to refine their approach to managing the hyponatremia associated with CNS infection. 

## 5. Conclusions

The risk of hyponatremia in patients with CNS infections is strongly associated with the infecting organism. Specifically, HSV infections demonstrated the highest risk while enteroviruses were associated with the lowest risk of hyponatremia. Half to two-thirds of patients with HSV CNS infection develop hyponatremia. However, the clinical syndrome alone can be moderately predictive of hyponatremia even without knowledge of the specific organism involved.

## Figures and Tables

**Table 1:** Patient characteristics and medication use by hyponatremia category.

	All Patients (*N* = 184) *n* (%)	Normonatremia (*N* = 112) *n* (%)	Hyponatremia (*N* = 72) *n* (%)
Median age (IQR) ^1^, years	38 (29–56)	34 (29–52)	46 (32–64)
Male sex (%)	89 (48.4)	51 (45.5)	38 (52.8)
Diabetes (%)	18 (9.8)	8 (7.1)	10 (13.9)
Heart failure (%)	3 (1.6)	2 (1.8)	1 (1.4)
eGFR ^2^ <60/mL/min/1.73m^2^	8 (4.4)	3 (2.7)	5 (6.9)
Encephalitis syndrome (%)	46 (25.0)	17 (15.2)	29 (40.3)
Acute kidney injury (%)	12 (6.5)	4 (3.6)	8 (11.1)
Acyclovir ^3^ (%)	79 (42.9)	41 (36.6)	38 (52.8)
Diuretics (%)	17 (9.2)	9 (8.0)	8 (11.1)
Angiotensin system inhibitor (%)	18 (9.8)	10 (8.9)	8 (11.1)
Antidepressants (%)	22 (12.0)	11 (9.8)	11 (15.3)
Antiepileptics (%)	10 (5.4)	4 (3.6)	6 (8.3)
Antipsychotics (%)	2 (1.1)	2 (1.8)	0 (0)

^1^ interquartile range, ^2^ estimated glomerular filtration rate, ^3^ 41% (32/79) had hyponatremia prior to acyclovir.

**Table 2:** Micro-organisms isolated in 99 patients by frequency.

Micro-Organism	Frequency	Percent
Enterovirus	36	36.4
Herpes simplex virus	19	19.2
Varicella zoster virus	15	15.2
*Streptococcus pneumoniae*	6	6.1
*Cryptococcus species*	6	6.1
*Neisseria meningitidis*	4	4.0
*Mycobacterium tuberculosis* ^1^	4	4.0
*Enterococcus faecium*	2	2.0
*Rickettsia species* ^1^	2	2.0
*Streptococcus agalactiae*	1	1.0
Influenza A virus ^1^	1	1.0
Respiratory syncytial virus ^1^	1	1.0
*Eikenella species* ^1^	1	1.0
*Leptospira interrogans*	1	1.0

^1^ organism isolated from blood or tissue samples by polymerase chain reaction or culture.

**Table 3:** Hyponatremia severity by organism category.

Serum Sodium	Viral *n* (%)	Bacterial *n* (%)	Fungal *n* (%)	None *n* (%)
Normal (≥135 mmol/L)	48 (67)	10 (48)	3 (50)	51 (60)
Mild (130 to 134 mmol/L)	16 (22)	7 (33)	2 (33)	28 (33)
Moderate or severe (≤129 mmol/L)	8 (11)	4 (19)	1 (17)	6 (7)
Total	72 (100)	21 (100)	6 (100)	85 (100)

**Table 4:** Hyponatremia severity by clinical syndrome.

Serum Sodium	Meningitis *n* (%)	Encephalitis *n* (%)
Normal (≥135 mmol/L)	95 (69)	17 (37)
Mild (130 to 134 mmol/L)	37 (27)	16 (35)
Moderate or severe (≤129 mmol/L)	6 (4)	13 (28)
Total	138 (100)	46 (100)

**Table 5:** Unadjusted logistic regression of hyponatremia.

Variable	Odds Ratio	(95% C.I.)	*p*-Value
Age, per 5-year increase	1.13	(1.04–1.23)	0.005
Male sex	1.34	(0.74–2.42)	0.34
Comorbidities			
Diabetes mellitus	2.10	(0.79–5.59)	0.14
eGFR <60 mL/min/1.73 m^2^	2.71	(0.63–11.7)	0.18
Cirrhosis ^1^	Not	estimable	
Heart failure ^1^	Not	estimable	
Acute kidney injury	3.38	(0.98–11.7)	0.054
Cerebrospinal fluid			
Leukocytes, per 100 × 10^6^ cells	1.01	(1.00–1.02)	0.24
Protein	1.21	(0.94–1.54)	0.14
Lactate	1.07	(0.97–1.19)	019
Glucose	0.95	(0.80–1.15)	0.59
Opening pressure ^1^	Not	estimable	
Organism			
None isolated	1.00	reference	0.029
Enterovirus	0.36	(0.14–0.92)	
Herpes simplex virus	3.25	(1.13–7.87)	
Varicella zoster virus	0.38	(0.10–1.43)	
*Streptococcus pneumoniae*	3.00	(0.52–17.3)	
*Cryptococcus* species	1.50	(0.29–7.87)	
*Neisseria meningitidis*	0.50	(0.05–5.01)	
*Mycobacterium tuberculosis*	1.50	(0.20–11.1)	
*Rickettsia* species	1.50	(0.09–24.8)	
Other ^1^	Not	estimable	
CNS infection syndrome			
Meningitis	1.00	reference	<0.001
Encephalitis	3.77	(1.87–7.58)	
Excessive hypotonic fluids ^1^	Not	estimable	
Taking medication(s) reported to cause hyponatremia	2.30	(1.17–4.54)	0.016
Diuretic ^2^	1.43	(0.53–3.90)	0.48
Angiotensin system inhibitor	1.28	(0.48–3.40)	0.63
Antidepressant	1.66	(0.68–4.50)	0.27
Antiepileptic	2.45	(0.68–9.02)	0.18
Antipsychotic ^1^	not	estimable	
Acyclovir ^3^	2.02	(0.58–7.04)	0.27

^1^ insufficient patients for estimates; ^2^ furosemide (13/184), thiazide (4/184), spironolactone (4/184); ^3^ patients with hyponatremia prior to treatment were excluded (*n* = 123).

**Table 6:** Multivariable logistic regression model of hyponatremia.

Variable	Odds Ratio	95% C.I.	*p*-Value
Age, per 5 year increase	1.08	(0.98–1.18)	0.12
Encephalitis syndrome ^1^	3.03	(1.43–6.39)	0.004

^1^ compared to meningitis syndrome as the reference group.

## Appendix A

**Table A1:** Patient characteristics and use of medications by clinical syndrome.

	All (*N* = 184) *n* (%)	Meningitis (*N* = 138) *n* (%)	Encephalitis (*N* = 46) *n* (%)
Median age (IQR) ^1^, years	38 (29–56)	33 (28–48)	56 (45–67)
Male sex (%)	89 (48.4)	66 (47.8)	23 (50.0)
Diabetes (%)	18 (9.8)	9 (6.5)	9 (19.6)
Heart failure (%)	3 (1.6)	2 (1.4)	1 (2.2)
Egfr ^2^ <60/mL/min/1.73 m^2^	8 (4.4)	5 (3.6)	3 (6.5)
Acute kidney injury (%)	12 (6.5)	6 (4.4)	6 (13.0)
Diuretics (%)	17 (9.2)	8 (5.8)	9 (19.6)
Angiotensin system inhibitor (%)	18 (9.8)	12 (8.7)	6 (13.0)
Antidepressants (%)	22 (12.0)	12 (8.7)	10 (21.7)
Antiepileptics (%)	10 (5.4)	4 (2.9)	6 (13.0)
Antipsychotics (%)	2 (1.1)	2 (1.4)	0 (0)

^1^ interquartile range, ^2^ estimated glomerular filtration rate.

## Appendix B

**Table A2:** Cerebrospinal fluid findings by infection type (median and interquartile range).

Cerebrospinal Fluid Parameter	Viral *n* = 72	Bacterial *n* = 21	Fungal *n* = 6	No Organism *n* = 85
Leucocytes (×10^6^/L)	187 (50–479)	662 (225–5970)	104 (22–238)	82 (16–218)
Polymorphs (×10^6^/L)	29 (10–74)	556 (142–5753)	20 (2–98)	10 (2–32)
Lymphocytes (×10^6^/L)	121 (24–355)	110 (46–337)	84 (20–140)	52 (10–164)
Protein (g/L)	0.7 (0.6–1.1)	2.6 (1.2–4.8)	1.1 (0.5–1.4)	0.7 (0.5–1.0)
Glucose (mmol/L)	3.5 (3.1–3.8)	1.6 (0.6–3.1)	3.0 (2.9–3.2)	3.6 (3.1–3.9)
Lactate (mmol/L)	2.3 (2.0–2.8)	8.0 (4.2–10.2)	2.9 (2.6–3.7)	1.9 (1.6–2.3)
